# Efficacy of eicosapentaenoic acid in inflammatory depression: study protocol for a match-mismatch trial

**DOI:** 10.1186/s12888-022-04430-z

**Published:** 2022-12-19

**Authors:** Klara Suneson, Filip Ängeby, Jesper Lindahl, Gustav Söderberg, Johanna Tjernberg, Daniel Lindqvist

**Affiliations:** 1 Department of Clinical Sciences Lund, Psychiatry, Faculty of Medicine, Lund University, 221 85 Lund, Sweden; 2 Office for Psychiatry and Habilitation, Psychiatric Clinic Helsingborg, Region Skåne, 252 23 Helsingborg, Sweden; 3 Office for Psychiatry and Habilitation, Psychiatric Clinic Lund, Region Skåne, 221 85 Lund, Sweden; 4 Department of Gastroenterology, Skåne University Hospital, 205 02 Malmö, Sweden; 5 Office for Psychiatry and Habilitation, Psychiatry Research Skåne, Region Skåne, 221 85 Lund, Sweden

**Keywords:** Major Depressive Disorder, Inflammation, N-3 PUFA

## Abstract

**Background:**

Most antidepressant treatment studies have included patients strictly based on the Diagnostic and Statistical Manual of Mental Disorders definition of Major Depressive Disorder (MDD). Given the heterogeneity of MDD, this approach may have obscured inter-patient differences and hampered the development of novel and targeted treatment strategies. An alternative strategy is ​​to use biomarkers to delineate endophenotypes of depression and test if these can be targeted via mechanism-based interventions. Several lines of evidence suggest that “inflammatory depression” is a clinically meaningful subtype of depression. Preliminary data indicate that omega-3 fatty acids, with their anti-inflammatory and neuroprotective properties, may be efficacious in this subtype of depression, and this study aims to test this hypothesis.

**Method:**

We conduct a match-mismatch-trial to test if add-on omega-3 fatty acid eicosapentaenoic acid (EPA) reduces depressive symptoms in patients with MDD and systemic low-grade inflammation. MDD patients on a stable antidepressant treatment are stratified at baseline on high sensitivity-C-reactive protein (hs-CRP) levels to a high-inflammation group (hs-CRP ≥ 3 mg/L) or a low-inflammation group (hs-CRP < 3 mg/L). Both groups receive add-on EPA (2 g per day) for 8 weeks with three study visits, all including blood draws. Patients and raters are blind to inflammation status. Primary outcome measure is change in Hamilton Depression Rating Scale score between baseline and week 8. We hypothesize that the inflammation group has a superior antidepressant response to EPA compared to the non-inflammation group. Secondary outcomes include a composite score of “inflammatory depressive symptoms”, quality of life, anxiety, anhedonia, sleep disturbances, fatigue, cognitive performance and change in biomarkers relating to inflammation, oxidative stress, metabolomics and cellular aging.

**Discussion:**

In this study we will, for the first time using a match-mismatch trial design, test if omega-3 is an efficacious treatment for inflammatory depression. If our study is successful, it could add to the field of precision psychiatry.

**Trial registration:**

This trial was registered May 8, 2017 on clinicaltrials.gov under the reference number NCT03143075

## Background

Among psychiatric disorders, depression is the number one cause of disability worldwide, and the overall second largest contributor to Years Lived with Disability [1]. Despite this, the pathophysiological mechanisms of depression are not fully understood and there are no clinically established biomarkers guiding treatment selection [2]. The poor remission rates with currently available treatments, most of which act on monoaminergic systems, suggest that there may be additional targets that could be therapeutically engaged [3].

The symptom heterogeneity of Major Depressive Disorder (MDD) may have hampered our understanding of the underlying biological mechanisms and slowed down the development of targeted pharmacological interventions. Therefore, we need biomarkers that could delineate more homogenous subsamples within this inclusive and heterogeneous diagnostic category, in order to test individualized treatments. Precision medicine, taking into account unique features of a patient’s pathology in selecting treatments, and improved patient stratification have been suggested as strategies to address issues of diagnostic heterogeneity [4]. A proposed, and widely studied, pathophysiological mechanism in depression is systemic low-grade inflammation. Available evidence suggests, however, that MDD is not an inflammatory disorder per se, but inflammation may rather contribute to the pathophysiology in some, but not all, cases of depression [5, 6]. The association between inflammation and depression has been studied extensively, and has been reviewed by our research group and others [6, 7]. In most published studies, MDD patients have higher mean levels of peripheral inflammatory markers compared to controls [8, 9], although there is a significant between-group overlap. Also, some patients treated with cytokine interferon-alpha (IFN-α) for hepatitis and other illnesses go on to develop depressive symptoms, further supporting the role of inflammation as a causative factor of depression [10]. Accumulating evidence suggests that some depressive symptoms are more inflammatory than others. Results from several large-scale studies show an association between inflammation and a specific depression symptom profile of anhedonia, lack of energy, sleep and appetite disturbances [11–14]. These symptoms overlap with so-called “sickness behavior”, seen in both humans and animals during states of infection [15].

It is still not clear whether alterations of immune markers seen in some depressed patients are caused by mechanisms in the periphery and/or in the central nervous system [16]. Animal studies have shown that depressive-like behavior may be mitigated by counteracting the effects of pro-inflammatory cytokines either in the blood or the brain [17]. Based on these reports and complementary clinical studies [18], interventions targeting inflammation in the periphery could be efficacious in treating psychiatric symptoms [17].

Several investigators have advocated for stratifying subjects based on inflammation status when designing clinical trials testing the antidepressant effect of anti-inflammatory drugs [17, 19, 20]. High-sensitivity C-Reactive Protein (hs-CRP) has been suggested as a candidate biomarker for this purpose [16, 21]. Hs-CRP can easily be obtained from a simple blood test and there are established cut-offs defining low-grade inflammation [22], as described in more detail below. Plasma hs-CRP may be a useful surrogate marker for both peripheral and central inflammation as it correlates with other inflammatory markers in blood and cerebrospinal fluid [16, 23]. Moreover, mean hs-CRP levels are increased in MDD compared to controls [24] and higher blood hs-CRP has been associated with changes in brain areas involved in motivation and motor activity; two important aspects of depressive symptomatology [25].

### Can omega-3 fatty acids be used to treat “inflammatory depression”?

Supplementation of polyunsaturated fatty acids (PUFAs) to individuals with cardiovascular disease decreases plasma levels of hs-CRP [26]. PUFAs omega-6 (n-6) and omega-3 (n-3) have several immune-modulating effects and are regarded as pro- and anti-inflammatory respectively [27]. They are found in cell membranes and compete for the same enzyme (delta 6-desaturase) for metabolization. Availability of n-6 in the cell membranes leads to the production of arachidonic acid (AA) and downstream pro-inflammatory agents such as prostaglandin E2. On the contrary, availability of n-3 PUFAs leads to more eicosapentaenoic acid (EPA) and docosahexaenoic acid (DHA) which in turn metabolize to anti-inflammatory agents resolvins and protectins [28]. These molecules exert their anti-inflammatory effects via various mechanisms including suppression of AA production. Hence, the balance between n-3 and n-6 PUFAs contribute to downstream formation of pro- or anti-inflammatory agents [7]. Humans have sparse or little de novo production of EPA and DHA [29] and are therefore dependent on dietary consumption of these compounds, which are readily available in fish and therefore called “marine PUFAs”. In addition to these mechanisms, other anti-inflammatory properties have also been attributed to n-3 fatty acids. These include (but are not limited to): 1. Reduced neutrophil and monocyte chemotaxis, 2. Reduction of adhesion molecule expression in the circulation and on immune cell surfaces, e.g. vascular cell adhesion molecules (VCAM) and intercellular adhesive molecules (ICAM), 3. Reduced prostaglandin production and 4. Suppressed proliferation of T-cells [as reviewed in 30]. The underlying mechanisms of these effects are still not completely known, but may involve n-3 fatty acids acting via cell surface and intracellular receptors regulating inflammatory cell signaling and gene expression patterns [30]. Besides anti-inflammatory effects, n-3 fatty acids may also have positive effects on leukocyte telomere length, telomerase activity, and other oxidative stress and cell aging markers [31]. However, more research is needed in order to confirm the effects of n-3 fatty acids on these biological processes and to determine any clinical relevance.

N-3 fatty acids have been found in some, but not all, studies to be superior to placebo in treating unipolar or bipolar depression, and the antidepressant effect of EPA has been greater than DHA [32]. Several reasons for caution have, however, been highlighted when interpreting these results, such as the small and possibly clinically irrelevant effect sizes and publication bias [33]. It is possible, given the anti-inflammatory properties of n-3 fatty acids, that this intervention is more efficacious in a subgroup of depression characterized by systemic low-grade inflammation. Consistent with this hypothesis, one Randomized Controlled Trial (RCT) showed that EPA (but not DHA) was effective in preventing IFN-α induced depression in patients with hepatitis C [34]. Consistent with a therapeutic effect of n-3 fatty acids in inflammatory depression, post hoc analyses from another RCT showed that EPA, but not DHA, was superior to placebo in reducing depressive symptoms but only in those depressed subjects with high inflammation markers at baseline [18]. Interestingly, effect sizes were large in this subgroup of inflammatory depression and increased with the number of inflammatory markers elevated. While these findings are suggestive of a specific effect of EPA on inflammatory depression, studies selecting patients a priori based on inflammatory markers are needed to confirm or refute this hypothesis.

### The main aims of this trial are to test if

1. Add-on EPA enriched n-3 has an antidepressant effect in patients with pre-treatment hs-CRP elevations.
2. The antidepressant effect is mediated by changes in inflammatory markers during the course of the study.
3. The current trial design with a priori stratification using inflammatory biomarkers is feasible, which could advance “precision psychiatry”.

## Methods

### Study design

This is a match-mismatch study with two parallel groups all receiving the same intervention. Participants are stratified according to inflammation status to a “low inflammation” or “high inflammation” group, based on high-sensitivity CRP (hs-CRP) levels before start of treatment. Participants and raters of the outcome measures are blind to group allocation during the study. Hs-CRP ≥ 3 mg/L is used to define the inflammation group based on recommendations from the Centers for Disease Control and the American Heart Association [22]. This cut-off has also been used in several depression studies to define low-grade inflammation [16, 18, 21]. Subjects will be instructed to postpone blood draw if they experience any signs of infection or sickness, and all study protocol deviations are noted. All subjects receive the same intervention of EPA 2 g/day added to their ongoing, stable medication.

### Primary and secondary outcomes

The primary outcome is absolute change in depressive symptoms between baseline and week 8, as measured with the Hamilton Depression Rating Scale 17-items (HAM-D-17). Secondary outcome measures are: Montgomery-Åsberg Depression Self-Rating Scale [35]; “inflammatory depressive symptoms”, defined as a composite score of Patient Health Questionnaire-9 [36] items #3 (sleep problems), #4 (lack of energy), #5 (appetite disturbance); overall function and quality of life using World Health Organization Disability Assessment Schedule [37]; anxiety symptoms using the Generalized Anxiety Disorder 7-item scale [38]; anhedonia using the Snaith-Hamilton Pleasure scale [39]; sleep disturbances using the Insomnia Severity Index [40]; fatigue using the Fatigue Severity Scale [41]; and speed of information processing, sustained attention and visual working memory using the Digit Symbol Coding Test [42].

### Overview and setting

The study was initiated in 2017 at the psychiatric clinic in Lund, Sweden. The study was approved by the ethical review board in Lund, Sweden (ref #2017/150). Amendments to the study protocol have been made, and approved, to improve recruitment and study procedures. To ensure proper collection and documentation of study results, records of study procedures and compliance with the approved protocol, the study is monitored by Clinical Studies Sweden, Forum South. Subjects are recruited via social media ads or clinical referrals.

At the screening visit, confirmation of MDD diagnosis according to DSM-5 and screening for potential comorbidities is carried out using the Mini International Neuropsychiatric Interview (MINI) [43]. As additional inclusion criteria, we chose cut-offs on the HAM-D-17 and the Clinical Global Impression scale (CGI) [44] in line with the proof-of-concept study from Rapaport et al. reporting that EPA might be efficacious in inflammatory depression [18]. Symptom rating scales and biomarkers are assessed at baseline, at weeks 4 and 8 (end of study).

### Eligibility criteria

Inclusion criteria:

1. Age 18–80 years
2. DSM-5 criteria fulfilled for current unipolar depressive episode (duration of symptoms > 4 weeks)
3. HAM-D-17 score ≥ 15
4. CGI severity score ≥ 3
5. Stable antidepressant or mood stabilizing treatment 6 weeks before study start
6. Subjects agree to not significantly modify their diet during the study

Exclusion criteria:

1. Medical illness that is serious or unstable and in the investigator's opinion could jeopardize response to treatment or interpretation of study results (e.g. malignancy, active or in remission < 1 year, insulin-dependent diabetes mellitus, active autoimmune disorder or inflammatory bowel disease)
2. Allergy to the study compounds
3. Current infection
4. Pregnancy or breast-feeding
5. Diagnosed psychotic or bipolar disorder, dementia, mental retardation, or individual who lack the ability to make an informed decision due to other conditions
6. Current electroconvulsive therapy
7. Anticoagulant treatment or known bleeding disorder
8. Current, serious suicidal or homicidal risk, in the judgment of investigator
9. Substance use disorder, except nicotine or caffeine, in the 3 months preceding the screening visit
10. Any medications that could confound the biomarker analyses, within 1 week of baseline or throughout the trial, including: regular intake of non-steroidal anti-inflammatory drugs (NSAIDs) or cyclooxygenase-2 (COX-2) inhibitors, or any use of oral steroids, immunosuppressants, chemotherapy, interferon. Patients will be instructed against intake of NSAIDs (including Aspirin) or COX-2 inhibitors in the 24 h preceding a visit including biomarker assessment visit
11. Intake of n-3 fatty acid supplementation for 3 consecutive days or more in the month preceding the screening visit
12. Initiation of psychotherapy during the last 4 weeks or plan to start psychotherapy during the study
13. Active participation with ongoing study visits in other clinical studies

### Study procedures

**Fig. 1:**
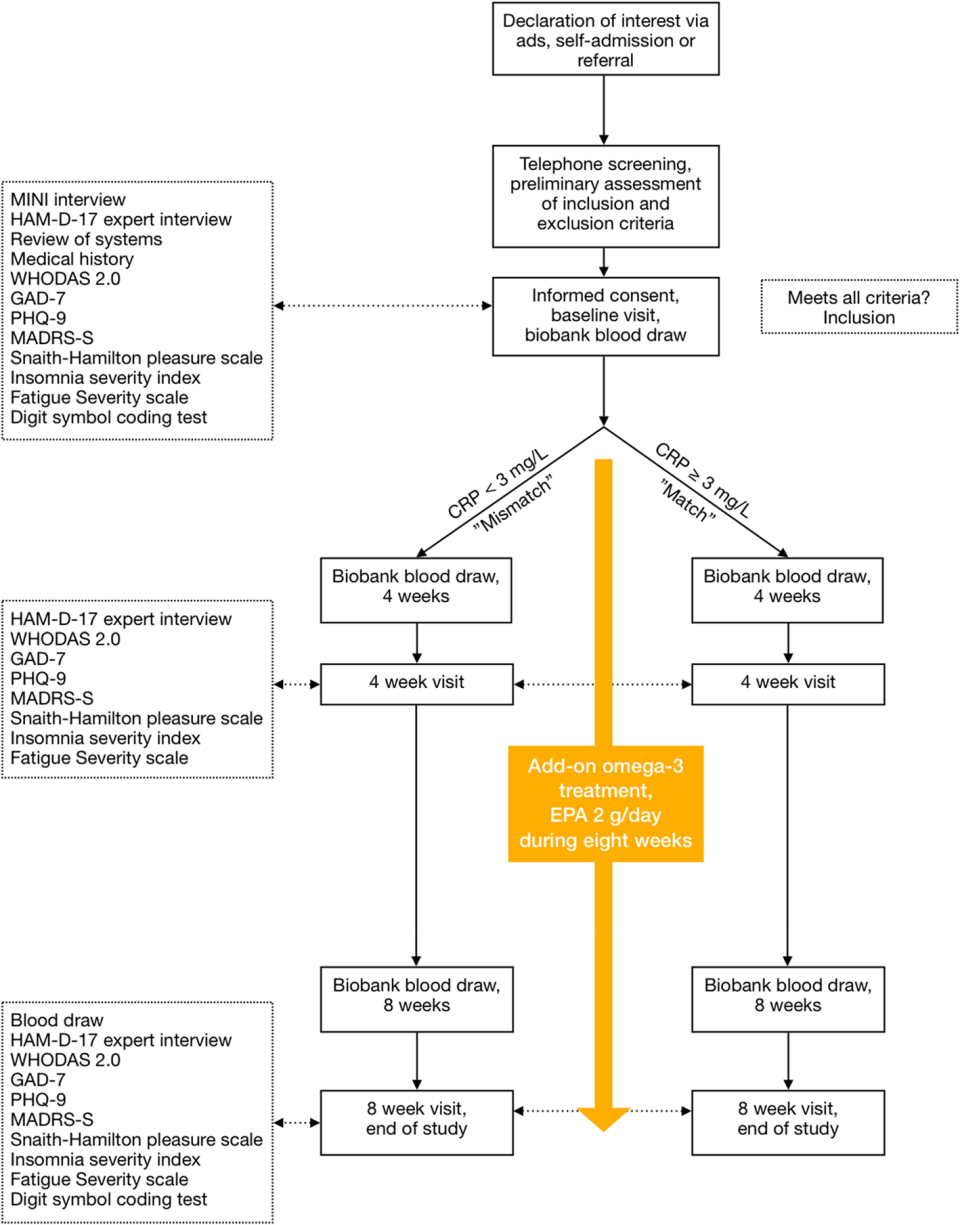
Schematic flow-chart describing the study protocol. Abbreviations: Hamilton Depression Rating Scale 17-item (HAM-D-17), World Health Organization Disability Assessment Schedule 2.0 (WHODAS 2.0), Mini International Neuropsychiatric Interview (MINI), Generalized Anxiety Disorder 7-item scale (GAD-7), Patient Health Questionnaire-9 (PHQ-9), Montgomery Åsberg Depression Rating Scale (MADRS-S), C-Reactive Protein (CRP), Eicosapentaenoic acid (EPA)

The study intervention is initiated at the baseline visit and continues for 8 weeks with additional study visits after four weeks and after eight weeks of treatment, as shown in Fig. 1.

Study eligibility is determined at the screening visit. The study physician conducts a review of systems and a complete medical history. Also, MINI interview, HAM-D-17 rating, and blood draw (hs-CRP) are assessed in order to determine eligibility. At baseline, week 4, and week 8 study visits, a larger blood draw (total of 36 ml each time) is carried out, and symptoms assessed using the rating scales outlined below. The Digit Symbol Coding test is completed at baseline and week 8.

Subjects eligible for inclusion start treatment with 2 g EPA per day*.* Subjects receive detailed instructions for how to take the capsules, and the number of remaining capsules are counted at each study visit to determine patient adherence. Subjects are provided with a “diary” and instructed to note the capsules taken (and any missed doses).

There is currently no consensus on the optimal EPA dose for treating depressive symptoms, with formulations ranging from approximately 1 g/day to 4 g/day [33], although doses as high as 9.6 g/day have been used in studies on depression with few and very mild side effects [45]. A recent meta-analysis on n-3 fatty acid supplementation in major depression showed similar numbers of adverse events in intervention and placebo groups [46]. Adverse events were predominantly gastrointestinal but psychological and other physical adverse events have also been reported [46]. There is a theoretical possibility that n-3 fatty acids might increase the risk of bleeding, although there is little clinical evidence supporting this link [47, 48]. Nevertheless, in the current study proposal we will exclude those subjects with a known bleeding disorder and those taking anticoagulants.

Subjects can, at any time, quit the study without any consequences for their standard clinical care. Disenrollment from the study might occur due to any of the following:

1. Physician’s clinical assessment
2. Subject preference
3. Emergent suicidality
4. Serious adverse events
5. Clinical evidence for need to increase or switch ongoing antidepressant
6. Worsening clinical depression ratings by > 20%

### Blood sample analyzes

Hs-CRP as obtained before intervention start is analyzed according to standardized clinical procedure.

In addition to hs-CRP to determine inflammation/non-inflammation group status, 6 blood tubes are drawn at baseline, week 4 and week 8 (6 tubes each time point); two serum tubes and 4 ethylenediaminetetraacetic acid (EDTA)-tubes. The serum tubes are centrifuged (2000 g × 10 min, 20 °C) within 30–60 min after the blood is drawn. Serum is aliquoted (distributed) manually into 16 REMP-tubes (300 μL) with 200 μL serum in each tube. Two of the EDTA-tubes are frozen in a -80 °C freezer. The other 2 EDTA-tubes are centrifuged (2000 g × 10 min, 20 °C) and the plasma is aliquoted manually into 16 REMP-tubes (300 μL) with 200 μL serum in each REMP-tube. The blood samples are then transported (in a container on dry ice) to the biobank for storage. After all subjects have been recruited, we will analyze blood biomarkers of potential relevance to the study treatment such as inflammation, oxidative stress, metabolomics, endothelial function, and cell aging.

### Statistical considerations

Based on the primary outcome of a difference in HAM-D-17 score between baseline and eight weeks of treatment, the minimum difference of the primary outcome we want to demonstrate is 3. In order to reject the null hypothesis that the population mean is equal between the two groups with a power of 0.80 using a T-test, we estimated, in the original sample size calculation, that 45 patients with the outcome in each group are needed (alpha = 0.05).

This is, to the best of our knowledge, the first trial of its kind. The original sample size calculation was based on information from the most similar patient group we could find with regards to the variance of the primary outcome in the intervention groups. An external statistician, not involved in any other parts of the study, conducted an interim check of the standard deviation of the primary outcome measure, and the number of subjects in each group. This was done after approximately 2/3 of the total sample had been recruited. Based on the interim check, a new sample size calculation was performed, estimating that a total of 96 subjects are needed (power = 0.8, alpha = 0.05).

The statistical model to be used to analyze our outcome measures is *mixed model repeated measures*, with patient as a random effect and treatment group, treatment week (week four or eight) and the interaction of treatment group and treatment week as fixed effects.

## Discussion

This is one of the first clinical trials in psychiatry to use a priori stratification of patients based on inflammatory biomarkers to test the efficacy of an anti-inflammatory intervention. There are, however, several previous studies that have used enrichment strategies to test the antidepressant effects of anti-inflammatories in a subgroup of inflammatory depression [49, 50]. These studies have, however, not included a non-inflammatory reference group, which might be necessary to answer the important clinical question if a biomarker can be used a priori to predict treatment response in a given depressed individual. Our study will be able to inform not only about the efficacy of this particular intervention (EPA) in a specific clinical population (inflammatory depression), but also test the feasibility of this novel match-mismatch study design in clinical psychiatric research.

The traditional RCT design is considered the gold standard for evidence-based medicine but has also been criticized for being expensive, biased in design, recruitment and data analysis, as well as testing general, broad feasibility rather than if the treatment is effective in certain, selected cases [51, 52]. Recent recommendations have been made to stratify subjects based on an a priori hypothesis, when assessing if a treatment might be efficacious in a certain subpopulation, as in this case of inflammatory depression [19]. Rapaport and colleagues found a benefit of using a combination of inflammatory markers to predict antidepressant treatment response to n-3 fatty acids [18]. Among individual inflammatory markers they found hs-CRP to be one of the strongest predictors of treatment response at baseline [18]. Even though serum hs-CRP is an established marker of inflammation both peripherally and centrally, levels may be affected by a variety of factors such as overweight and old age, as well as infections, or undiagnosed acute or chronic inflammatory processes. This was taken into consideration when designing the study, specifically when defining our exclusion criteria. Since all participants in this match-mismatch study receive the same active treatment and instead are stratified based on levels of inflammation, potential confounders such as off-target effects and adverse events might have less of an impact. The design makes it possible, in comparison to traditional RCTs, to discern whether the treatment is broadly effective in all patients or more effective in a smaller group with high inflammation [17].

For this study, we chose an approach where the primary outcome measure is an expert assessment scale, the HAM-D-17. We decided, however, to also add several secondary outcome measures including self-ratings, objective measures of cognitive performance and blood biomarkers. Our primary outcome measure, change in the HAM-D-17 score, was chosen mainly since it would facilitate comparison of our results with previous studies. The HAM-D-17 has been commonly used in clinical work and psychiatric research since its introduction in 1960 [53]. It has long been considered the gold standard but it has also been criticized with regards to poor inter-rater and retest reliability [54]. The HAM-D might also have other shortcomings including that several items measure de facto antidepressant side effects (*e.g.* insomnia, weight loss) which, some have argued, should result in the selection of shorter, unidimensional rating scales in future clinical trials [55]. In this study, we use secondary outcome measures related to anhedonia and other symptoms previously found to be associated with inflammation [11, 12, 14] that we believe could address some of these concerns.

Secondary outcome measures will be analyzed using biomarkers, representing the various pathways on which the n-3 fatty acids have its effect. Besides anti-inflammatory properties, n-3 fatty acids are known to exert antioxidative effects [56], hence we are planning to assess biomarkers of oxidative stress, such as F2-isoprostane, 8-OH 2-deoxyguanosine and glutathione. Other pathways associated with low-grade chronic inflammation and old age are cell aging parameters and endothelial biomarkers such as leukocyte telomere length, telomerase activity, and ICAM, VCAM [30, 31].

Furthermore, we aim to investigate potential downstream effects of low-grade inflammation such as activation of the kynurenine pathway (KP) and formation of its neuroactive metabolites. Elevated levels of TNF-α and the ratio of kynurenine/tryptophan, indicating inflammation-induced activation of the KP, have recently been found in a subgroup of depression with more severe anhedonia and poorer treatment response [57]. Lastly, also potential up-stream mechanisms, triggering inflammation, will be assessed through measuring potential proxy markers of altered gut-brain axis activity [58] and gut permeability, previously associated with depression [59]. In secondary analyzes, we also aim to investigate associations between EPA treatment response and metabolic alterations by measuring biomarkers such as leptin, adiponectin, lipids, glucose, and metabolomics.

Our study comes with several limitations, including that we have not obtained any structured information on dietary habits before and during the study. Yet, we ask subjects not to make any major changes in their eating habits. Moreover, the recruitment phase has been prolonged due to the COVID-19 pandemic and an initial lack of referrals from other health-care facilities. We have since gained much interest via ads on social media which has improved our overall recruitment procedures.

## Data Availability

Not applicable.

## Abbreviations

AA: Arachidonic acid
CGI: Clinical Global Impression scale
COX-2: Cyclooxygenase-2
DSM: Diagnostic and Statistical Manual of Mental Disorders
DHA: Docosahexaenoic acid
EPA: Eicosapentaenoic acid
EDTA: Ethylenediaminetetraacetic acid
GAD-7: Generalized Anxiety Disorder 7-item scale
HAM-D-17: Hamilton Depression Rating Scale 17-items
hs-CRP: High sensitivity C-Reactive Protein
ICAM: Intercellular adhesive molecules
KP: Kynurenine pathway
MDD: Major Depressive Disorder
MINI: Mini International Neuropsychiatric Interview
MADRS-S: Montgomery Åsberg Depression Rating Scale
NSAIDs: Non-steroidal anti-inflammatory drugs
n-3: Omega-3
n-6: Omega-6
PHQ-9: Patient Health Questionnaire-9
PUFAs: Polyunsaturated fatty acids
RCT: Randomized Controlled Trial
VCAM: Vascular cell adhesion molecules
WHODAS 2.0: World Health Organization Disability Assessment Schedule 2.0
